# Single-Port Robot assisted partial cystectomy for urachal adenocarcinoma

**DOI:** 10.1590/S1677-5538.IBJU.2024.0379

**Published:** 2024-07-25

**Authors:** Sij Hemal, Sina Sobhani, Kevin Hakimi, Shilo Rosenberg, Inderbir Gill

**Affiliations:** 1 University of Southern California Norris Comprehensive Cancer Center Institute of Urology Los Angeles CA USA Institute of Urology, Norris Comprehensive Cancer Center, University of Southern California, Los Angeles, CA, USA

## Abstract

**Objective:**

We present a novel technique to perform single-port (SP) robot-assisted partial cystectomy with excision of the urachal remnant and bilateral pelvic lymph node dissection for urachal adenocarcinoma ( 1 – 7 ).

**Materials and Methods:**

A 41-year-old male presented to the clinic for multiple episodes of hematuria and mucousuria. Office cystoscopy revealed a small solitary tumor at the dome of the bladder, with a diagnostic bladder biopsy revealing a tubule-villous bladder adenoma. Cross-sectional imaging of the chest/abdomen/pelvis revealed a 4.5 cm cystic mass arising from the urachus without evidence of local invasion and metastatic spread. He underwent SP robotic-assisted partial cystectomy with excision of the urachal remnant and bilateral pelvic lymph node dissection. Surgical steps include: 1) peritoneal incision to release the urachus and drop bladder 2) identification of urachal tumor 3) intraoperative live cystoscopic identification of bladder mass and scoring of tumor margins using Toggle Pro feature 4) tumor excision with partial cystectomy 5) cystorrhaphy 6) bilateral pelvic lymph node dissection 7) peritoneal interposition flap to mitigate lymphocele formation.

**Results:**

Surgery was successful, with no intraoperative complications, an operative time of 100 minutes, and estimated blood loss of 20 mL. The patient was discharged on post-op day one, and the Foley catheter removed one week after surgery. Final pathology revealed a 7.5 cm infiltrating urachal muscle-invasive adenocarcinoma of the bladder (pT2b). Negative surgical margins were achieved.

**Conclusions:**

Single-port robot-assisted partial cystectomy for urachal adenocarcinoma is safe and can achieve equivalent oncologic outcomes to the standard of care with minimally invasive and open techniques.

**Available at:** http://www.intbrazjurol.com.br/video-section/20240379_Sobhani_et_al

**Int Braz J Urol. 2024; 50 (Video #10): 659-60**
